# Which component of mechanical power is most important in causing VILI?

**DOI:** 10.1186/s13054-020-2747-4

**Published:** 2020-02-05

**Authors:** John J. Marini, Patricia R. M. Rocco

**Affiliations:** 1 Division of Pulmonary and Critical Care Medicine, Regions Hospital, University of Minnesota, MS11203B, 640 Jackson St., Minneapolis/St. Paul, MN 55101 USA; 2 Laboratory of Pulmonary Investigation, Carlos Chagas Filho Biophysics Institute, Federal University of Rio de Janeiro, Av. Carlos Chagas Filho 373, Ilha do Fundão, Rio de Janeiro, 21942-902 Brazil

**Keywords:** Ventilator-induced lung injury, Inflation energy, Ventilating power, ARDS

## Background: energy, power, and VILI

Repeated applications of tidal energy inflict lung damage (VILI) when stress and strain exceed the limits of tissue tolerance. Inflation work and energy are the products of pressure and volume, which are loosely associated with stress and strain, respectively. Three major pressure components contribute to tidal inflation energy: flow resistive pressure, tidal elastic (driving) pressure, and the static elastic pressure baseline set by PEEP [1, 2].

Although total inflation power, i.e., the energy per cycle multiplied by ventilating frequency, has been causally linked to VILI risk [3], not all combinations of the three pressure components of energy and frequency that sum to the same total power value are equally hazardous. For injury to occur, a second requirement (apart from the raw power total) is a precondition; enough strain *per cycle* must be imposed to sufficiently distort lung parenchyma or disrupt vulnerable elements of the extracellular matrix that comprise the interdependent supporting framework for the alveolar network. The strain actually experienced at the tissue level depends on local force-amplifying influences such as geometrical stress focusing and rate of parenchymal expansion [4]. Duration of exposure (which determines the cumulative number of high stress cycles) is clearly of importance once the injuring strain threshold has been breached. Finally, whether or not a given pattern of ventilation injures also depends on lung capacity and innate vulnerability of lung tissues to the applied mechanical stress. Thus, a healthy lung can withstand power loads much greater than do the relatively fragile but functional alveoli that make up the “baby lung” of ARDS [5].

## Sub-components and thresholds of tidal energy

Regarding the monitored variables of ventilation that relate to total inflation pressure, active debate has developed as to whether the element with most influence is end-inspiratory static (plateau) pressure or its difference from PEEP, known as the driving pressure (ΔP) [6–8]. PEEP bears a U-shaped relationship to VILI risk, with low levels tending to reduce atelectasis and distribute stress [9, 10]. On the other hand, high PEEP sets an elevated platform from which widespread tidal overstretching of the parenchyma as well as hemodynamic compromise become increasingly likely [10]. Finally, neither PEEP, plateau pressure, nor their difference (ΔP) reflect the dynamics and dissipated energy of the tidal cycle.

**Fig. 1:**
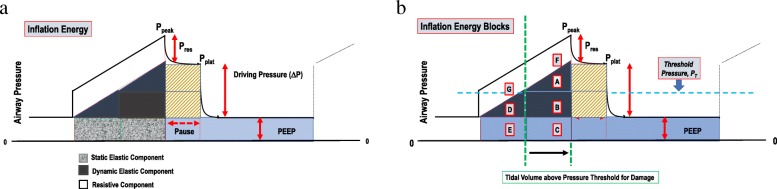
**a** *Left Panel*: Elastic and flow resistive components of the total energy per tidal cycle during passive constant flow. **b** *Right Panel*: An arbitrarily assumed threshold for damaging pressure divides total tidal energy into subcomponent blocks below and above the boundary that separates potentially non-injurious (blocks G, D, and E) from injurious contributors (blocks F, A, B, and C), respectively. Simple algebraic estimates for each can be developed from clinical data using pre-specified parameters of mechanics or empirically observed measurements at the bedside. Example: total power = *f* × [*A* + *B* + *C* + *D* + *E* + *F* + *G*] = *V*’_E_ × (*RV*’ + *V*_T_/2*c* + PEEP) or alternatively: (*V*’_E_/2) × (2 *P*_peak_ − *P*_plat_ + PEEP). Total *Dynamic Excessive* Power = *f* × [*A* + *B* + *C*] = *V*’_E_ × {1 + [(PEEP − *P*_T_) × *c*]/*V*_T_} × ½ [(*V*_T_/*c*) + PEEP + *P*_T_] or alternatively: *V*’_E_ × [(*P*_plat_ − *P*_T_)/(*P*_plat_ − PEEP)] × [(*P*_plat_ + *P*_T_)/2)]_._ *Abbreviations*: *R* = Resistance; *c* = Compliance

While energy clearly must be expended to produce damaging strain, uncertainty exists regarding which component of the applied energy per inflation cycle deserves closest attention. As implied by the earlier discussion, the “energy envelope” of tidal inflation encloses a total pressure-volume area defined by the combination of the flow-resistive, tidal elastic and PEEP-associated sectors that comprise it (Fig. 1a). Although flow-resistive energy largely dissipates in pushing gas past the endotracheal tube and through conducting airways, rapid movements of parenchymal tissues induce greater strains than slower ones—both during inflation and deflation [4, 11, 12].

The influence of PEEP-related inflation energy often has been discounted as well, using the reasoning that the pressure baseline is eventually recovered at the end of the deflation phase [8, 13]. Whatever the perceived wisdom of that argument, PEEP undeniably elevates volume and strain over the resting values of their fully relaxed states. In so doing, raising PEEP requires that each inflation volume increment is achieved at higher absolute pressure and with greater energy input. The driving pressure (tidal elastic) component of inflation energy parallels the amplitude of tidal volume and only quantifies *incremental* work due to raising elastic pressure above baseline PEEP.

It must be recognized that VILI may manifest in various ways: e.g., as inflammation, cellular disruption, or focal hyperinflation, with different tidal pressure components contributing unequally to each [9, 10]. For an individual lung, thresholds for stress, strain, energy, and power must exist, below which their applications pose little threat of whichever kind (Fig. 1a). Such thresholds are conditioned by the underlying integrity of the tissue elements, by the stage and extent of injury, and by the topographical position of the individual lung unit under consideration (local stress environment). In concept, such thresholds migrate to lower or higher levels as disease progresses or recedes.

While input of cumulative total energy and total power have been experimentally demonstrated to be linked to VILI [3, 14, 15], exactly which delivered component or subcomponent of power best correlates with damage has not been clearly identified. For example, it might be argued that only energy that relates to driving pressure or total elastic pressure (the product of volume and *absolute* alveolar pressure, ΔP + PEEP) would best track stretch and VILI risk. Alternatively, the total kinetic energy applied in excess of PEEP (the product of volume and the sum of ΔP plus flow resistive pressure) might hold equal interest regarding inflammatory changes. Furthermore, if we assume the existence of a pressure-strain threshold for damage and take it into consideration, perhaps only energy applied in excess of that level would be VILI-relevant. A breakdown of total tidal energy by threshold pressure gives rise to clinically measurable “blocks” of energy during passive inflation with constant flow (Fig. 1b). As the search to define the safety limits of power application proceeds, one or more of these energy sub-blocks hold potential to sharpen the precision of setting an upper target for lung-protecting power.

## Estimating components of inflation energy

In theory, with resistance and compliance known and a threshold assumed, relatively simple algebraic formulae could estimate energy components (“blocks”) of research or clinical interest that are defined by pressure-volume areas during passive inflation with constant flow. These formulae use reasonable simplifying assumptions and a geometric approach to analysis (Fig. 1b). The theoretical impact of a proposed change in ventilation on the components of this energy/power complex could then be numerically estimated before they are actually made. Using similar principles when only *already measured* pressures, flow, volume, and frequency and are at hand, a comparable rationale and method produce condensed formulae that estimate these same energy components, so as to characterize the pattern already in use or to serially track the evolution and resolution of the ergo-trauma determinants of greatest interest (Fig. 1b).

## Data Availability

Not applicable.

## Abbreviations

*P*_peak_: Peak pressure
*P*_res_: Resistive pressure
*P*_plat_: Plateau pressure
*P*_T_: Threshold pressure
Δ*P*: Driving pressure
*V*_T_: Tidal volume
*V*_E_: Minute ventilation
C: Compliance
V: Airflow
R: Airflow resistance
